# Graphitic Carbon Nitride-Based Heterojunctions for Photocatalytic Water Purification: Mechanisms, Fabrication, and Applications

**DOI:** 10.3390/nano16211363

**Published:** 2026-10-26

**Authors:** Jinhua Zhuang, Heshan Cai, Sibo Ding, Zekai Liu, Haopeng Liao, Haoze Jiang, Yuyue Luo, Chaoming Liang, Hang Zhang, Songran Guan, Daguang Li

**Affiliations:** 1 School of Environmental and Chemical Engineering, Foshan University, Foshan 528200, China; 2 College of Light Chemical Industry and Materials Engineering, Shunde Polytechnic University, Foshan 528300, China

**Keywords:** graphitic carbon nitride, heterojunction, photocatalysis, pollutants, water purification applications

## Abstract

As a metal-free, visible-light-responsive two-dimensional (2D) layered semiconductor, graphitic carbon nitride (g-C_3_N_4_) has been extensively studied for photocatalytic water purification owing to its high chemical stability, facile synthesis, and low cost. However, single-phase g-C_3_N_4_ still suffers from bottlenecks including rapid photogenerated electron–hole pair recombination, low visible-light utilization efficiency, insufficient specific surface area, and a limited number of exposed surface active sites, which severely restrict its practical photocatalytic performance. Rational construction of heterojunctions with matched band alignment and intimate interfacial contact serves as an effective strategy to boost the photocatalytic activity of g-C_3_N_4_. This review introduces the structural characteristics and photocatalytic mechanisms of g-C_3_N_4_, summarizes the primary types of g-C_3_N_4_-based heterojunctions along with their charge transfer pathways, and highlights recent research progress in emerging contaminant degradation, pathogenic microbial disinfection, and reduction of high-valence and highly toxic heavy-metal ions for water decontamination. The review closes with critical perspectives on the rational design and practical engineering prospects of g-C_3_N_4_-based heterojunctions. By bridging heterojunction mechanisms with water-purification applications and engineering considerations, this review is expected to guide the rational design of high-performance g-C_3_N_4_-based heterojunctions and accelerate their translation toward practical water purification.

## 1. Introduction

In recent years, industrial and municipal discharges have rendered aquatic pollution increasingly complex, featuring emerging contaminants, pathogenic microorganisms, and heavy metal ions. Emerging contaminants, including antibiotics, endocrine-disrupting chemicals (EDCs), and microplastics, are continuously discharged into the environment, exhibit complex migration-transformation behaviors and pose uncertain ecological risks; highly toxic heavy metal species and microbial pathogens pose severe chemical toxicity and biosafety hazards, respectively [1]. Conventional water treatment technologies (e.g., adsorption, coagulation, and biological treatment) offer limited removal capability toward recalcitrant and highly toxic pollutants, frequently accompanied by incomplete mineralization, secondary pollution risks, and poor adaptability toward complex matrices, failing to meet demands for deep-level water purification [2,3]. Consequently, developing efficient, green, and sustainable water purification technologies has become an urgent environmental priority [4].

Photocatalytic technology utilizes solar energy to drive redox reactions and represents an environmentally benign pathway for pollutant degradation, microbial disinfection, and high-valence metal ion reduction. As an archetypal metal-free polymeric semiconductor, g-C_3_N_4_ features a bandgap of ~2.7 eV, visible-light responsiveness, high chemical stability, earth-abundant raw materials, and facile synthesis, rendering it one of the most intensively studied materials for photocatalytic water purification [5]. Nevertheless, pristine g-C_3_N_4_ is restricted by inherent performance bottlenecks, and single-component systems struggle to fulfill practical water treatment requirements. Coupling g-C_3_N_4_ with band-matched semiconductors to engineer heterojunctions can effectively manipulate carrier migration pathways and improve charge separation efficiency, representing an effective strategy for enhancing its photocatalytic activity [6,7]. Since the landmark report by Wang et al. [8] establishing graphitic carbon nitride as a visible-light-active metal-free polymeric semiconductor, g-C_3_N_4_-based systems have garnered immense interest. Driven by these advantages, g-C_3_N_4_-based heterojunctions offer substantial research value and broad application potential in water decontamination [9].

**Figure 1:**
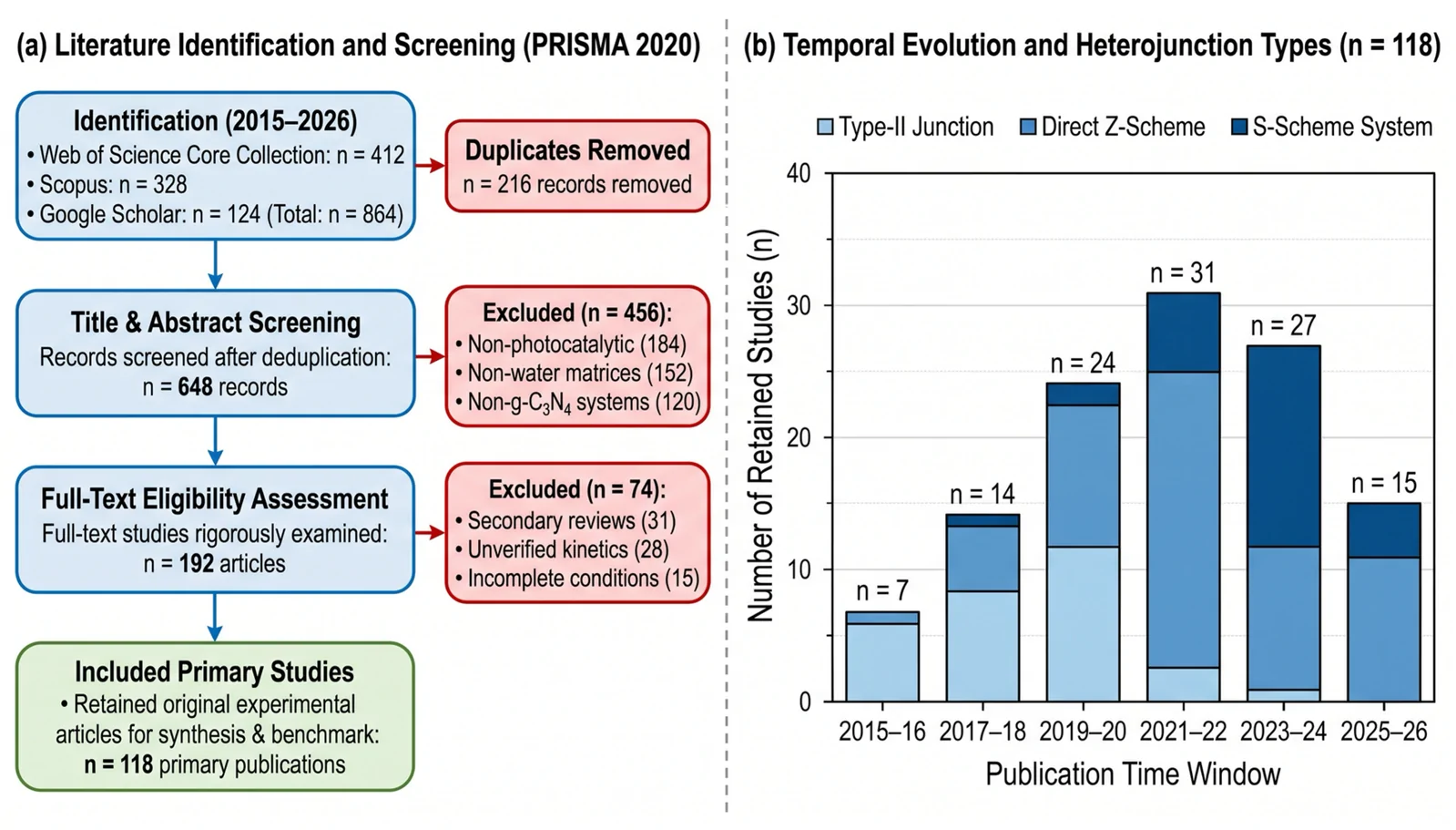
Transparent literature methodology and bibliometric distribution for g-C_3_N_4_-based heterojunctions in water remediation (2015–2026): (**a**) PRISMA 2020 flow diagram: 864 records identified (Web of Science Core Collection, 412; Scopus, 328; Google Scholar, 124), 216 duplicates removed, 648 records screened, 456 excluded (184 non-photocatalytic, 152 non-water matrices, 120 non-g-C_3_N_4_ systems), 192 full-text articles assessed, 74 excluded (31 secondary reviews, 28 unverified kinetics, 15 incomplete conditions), and 118 primary studies included; and (**b**) temporal evolution and heterojunction-configuration distribution (Type-II, direct Z-scheme, and S-scheme) across the 118 included studies.

This review systematically summarizes recent advances in g-C_3_N_4_-based heterojunctions for photocatalytic water purification. The literature selection followed the PRISMA 2020 guidelines (Figure 1). A total of 864 records published between 2015 and 2026 were identified from the Web of Science Core Collection (*n* = 412), Scopus (n = 328), and Google Scholar (n = 124). After removing 216 duplicates, 648 records were screened by title and abstract, of which 456 were excluded (184 non-photocatalytic studies, 152 studies not involving water matrices, and 120 studies not based on g-C_3_N_4_). The remaining 192 articles were assessed in full text, and 74 were excluded (31 secondary reviews, 28 studies with unverified kinetics, and 15 studies reporting incomplete experimental conditions), leaving 118 primary experimental studies for quantitative synthesis and mechanistic benchmarking. The search string applied in the Web of Science and Scopus was ((“graphitic carbon nitride” OR “g-C_3_N_4_” OR “C_3_N_4_”) AND (heterojunction OR “type-I” OR “type-II” OR “Z-scheme” OR “S-scheme”) AND (photocatal) AND (water OR wastewater OR pollutant OR degradation OR disinfection OR antibacterial))). Google Scholar was searched with the simplified string “graphitic carbon nitride” AND heterojunction AND photocatalysis AND (water OR wastewater). The search was limited to English-language journal articles; reviews, conference abstracts, editorials, and studies without original experimental photocatalytic data were excluded. The discussion begins with the intrinsic structural characteristics and basic photocatalytic mechanisms of g-C_3_N_4_, followed by an in-depth analysis of the electronic transfer pathways of distinct heterojunction configurations and a systematic categorization of mainstream fabrication protocols, including chemical vapor deposition, hydrothermal/solvothermal methods, thermal calcination, In Situ Polymerization and Deposition–Precipitation and Electrostatic/Ultrasonication Assembly and Photodeposition. Practical implementations in degrading emerging pollutants, inactivating pathogens and reducing toxic heavy metals are then reviewed, and constructive perspectives and future recommendations are provided in closing to serve as valuable references for subsequent investigations in this field [10]. The g-C_3_N_4_-based photocatalysis has been surveyed from multiple angles in recent years, covering homojunctions [11], carbon nitride/graphene composites for emerging pollutant degradation [12], mechanistic comparisons of type-II, Z-scheme and S-scheme heterojunctions [13], Z-scheme catalysts for microplastics mineralization [14], morphology-controlled g-C_3_N_4_/metal oxide nanostructures [15], pharmaceutical and antibiotic degradation [16,17,18], g-C_3_N_4_/Bi-based Z-scheme systems for the removal of pharmaceuticals and personal care products [19], and S-scheme heterojunctions for U(VI) reduction [20]. Distinct from existing reviews that survey single applications or material families, the novelty of this review is anchored in establishing an integrative ‘mechanism-fabrication-engineering’ framework. Specifically, this review uniquely provides: (i) an uncompromising critical assessment of the experimental validity of claimed Type-I, Type-II, Z-scheme, and S-scheme charge-transfer pathways; (ii) a multi-dimensional benchmarking matrix across fabrication routes balancing interface quality with industrial scalability; and (iii) a pragmatic water-treatment assessment that integrates practical water-matrix interferences, reaction byproducts, and reactor modeling into catalytic evaluations.

## 2. Structure and Photocatalytic Mechanism of g-C_3_N_4_

**Figure 2:**
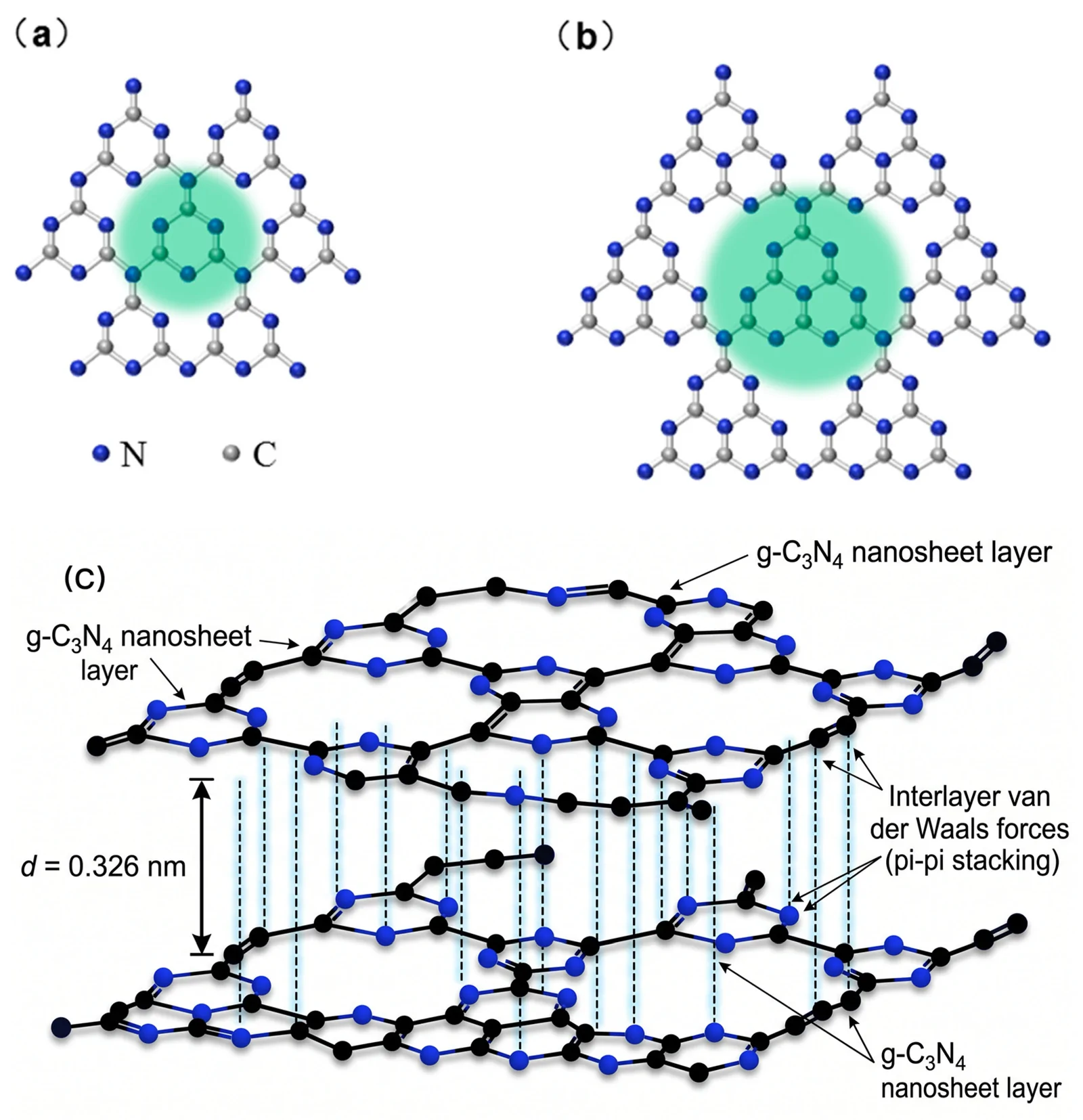
Schematic structures of g-C_3_N_4_: (**a**) triazine unit; (**b**) heptazine unit. Reproduced from Ref. [34] under CC BY 4.0 license. (**c**) 3D layered structure illustrating the interlayer distance (d ≈ 0.326 nm) and interlayer van der Waals interactions.

g-C_3_N_4_ is a 2D layered non-metallic semiconductor composed of carbon and nitrogen atoms bonded via sp^2^ hybridization to establish an extended, highly conjugated π-electron network. Its basic structural units comprise triazine (C_3_N_3_) or heptazine/tri-s-triazine (C_6_N_7_) motifs, which periodically assemble into graphite-like planar networks held together by interlayer van der Waals forces (Figure 2). g-C_3_N_4_ exhibits notable chemical and thermal stability [21]. It features a bandgap of ~2.7 eV, enabling optical absorption in the blue-edge visible region (λ ≤ 460 nm). Its conduction band (CB) potential is positioned at approximately –1.1 V (vs. NHE, pH = 7), whereas the valence band (VB) edge lies at ~+1.6 V [22]. It is hereby clarified that all band-edge potentials (CB and VB) discussed in this section are normalized to pH = 7 and expressed versus the normal hydrogen electrode (NHE), unless explicitly stated otherwise. Because band-edge positions shift with pH and measurement method, reported CB potentials of g-C_3_N_4_ span a range (approximately −1.1 to −1.63 V vs. NHE); values quoted in subsequent sections follow the conditions reported in the respective original studies (see Section 5.3) [23]. Complementary to experimental electrochemical determinations (e.g., Mott-Schottky analysis and ultraviolet photoelectron spectroscopy), density functional theory (DFT) calculations provide a powerful first-principles approach to accurately determine band edge potentials [24,25]. By constructing periodic slab models and referencing the total electrostatic potential to the absolute vacuum level (E_vac_), the valence band maximum (VBM) and conduction band minimum (CBM) can be extracted directly from the projected density of states (PDOS). Given that conventional generalized gradient approximations (GGA-PBE) systematically underestimate the semiconductor bandgap, hybrid functionals (such as HSE06) or PBE+U schemes are widely implemented to correct self-interaction errors, yielding band edges and work functions that align closely with experimental values vs. NHE (E_NHE_ = −E_vac_ − 4.44 V at pH = 0, corrected to pH = 7 via the Nernstian shift of −0.059 V/pH). Furthermore, DFT slab calculations can resolve plane-dependent surface dipoles, work-function discrepancies, and interfacial charge redistribution, providing quantitative theoretical verification for contact-induced band bending and internal electric-field (IEF) alignment across g-C_3_N_4_ heterojunctions [26].

Under visible-light irradiation, when incident photon energy (hν) equals or exceeds the bandgap (Eg) of g-C_3_N_4_, intrinsic interband optical absorption occurs. Valence band electrons absorb photons and cross the forbidden band into the conduction band, leaving corresponding holes behind to generate photogenerated electron–hole (e^−^/h^+^) pairs [27,28]. Subsequently, photogenerated electrons and holes migrate from the bulk toward surface catalytic sites. Upon reaching the surface, CB electrons transfer to adsorbed electron acceptors (e.g., dissolved O_2_ or H^+^) to drive reduction reactions; dissolved oxygen is reduced to superoxide radicals (O_2_^•−^), or high-valence metal ions (e.g., Cr(VI)) are reduced directly; simultaneously, VB holes can directly oxidize surface-adsorbed organic pollutants [29]. However, pristine g-C_3_N_4_ suffers from rapid carrier recombination, low intrinsic carrier mobility, and short diffusion lengths, causing a considerable fraction of charge carriers to recombine within the bulk or on the surface before participating in surface catalysis [30]. In addition, bulk g-C_3_N_4_ easily undergoes interlayer stacking, yielding low specific surface areas and few exposed active sites, which gives rise to mass-transfer limitations and interfacial charge-transfer resistance; its optical absorption range and surface redox kinetics also leave room for enhancement. To overcome these limitations, building g-C_3_N_4_-based heterojunctions has emerged as a primary modification strategy [31]. Coupling g-C_3_N_4_ with complementary band-matched semiconductors establishes interfacial built-in electric fields and staggered band alignments, driving photogenerated electrons and holes in opposing directions to realize spatial separation, thereby suppressing recombination and extending carrier lifetimes [32]. In addition, rational selection of narrow-bandgap components or structural tailoring synchronously expands optical absorption and multiplies accessible reaction sites [33].

## 3. Main Types of g-C_3_N_4_-Based Heterojunctions

**Figure 3:**
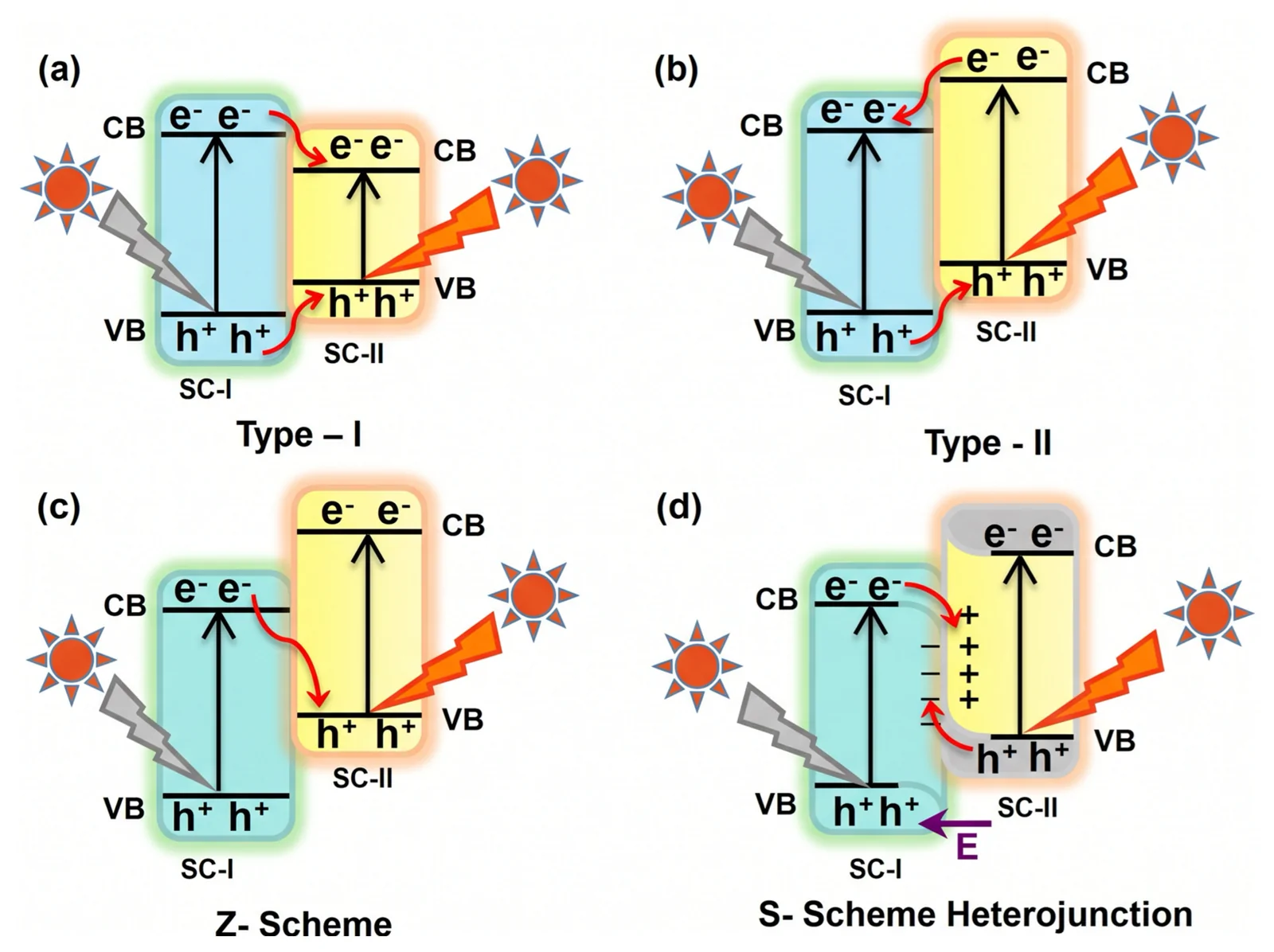
Carrier separation processes of different heterojunctions under light irradiation: (**a**) Type-I; (**b**) Type-II; (**c**) Direct Z-scheme; and (**d**) S-scheme heterojunctions. Solid black upward arrows denote photogeneration and excitation of electron–hole pairs; red curved arrows indicate charge transfer and interfacial recombination pathways; and the purple arrow represents the built-in electric field (IEF) across the interface.

A heterojunction consists of two or more distinct semiconductor materials in intimate contact at their interface. When g-C_3_N_4_ couples with secondary semiconductors to construct heterojunctions, band alignment and interfacial contact dictate carrier migration and recombination kinetics, thereby tuning photocatalytic performance. Depending on band alignments, interfacial charge transfer directions, and carrier separation pathways, g-C_3_N_4_-based heterojunctions are classified into Type-I, Type-II, Z-scheme, and S-scheme configurations (Figure 3), where SC-I and SC-II denote Semiconductor I and Semiconductor II, respectively.

### 3.1. Type-I Heterojunction

In a Type-I heterojunction (Figure 3a), both the CB and VB edges of SC-II are located within the bandgap of SC-I, meaning SC-II exhibits a narrower bandgap. Under irradiation, both SC-I and SC-II absorb photons exceeding their bandgaps, generating CB electrons and VB holes [35]. Driven by energy level offsets, electrons in the higher-energy CB of SC-I migrate to the lower-energy CB of SC-II; concurrently, holes in the VB of SC-I transfer to the higher-energy VB of SC-II [36]. Before interfacial contact, the isolated semiconductors (SC-I and SC-II) possess distinct work functions and Fermi levels (E_F_). Upon intimate contact, a spontaneous diffusion of electrons occurs across the interface driven by the electrochemical potential gradient until a unified Fermi level is established at thermodynamic equilibrium. This interfacial charge redistribution establishes a localized space-charge region and weak interfacial band bending. Under irradiation, despite this thermal equilibration, photogenerated electrons and holes are thermodynamically driven to accumulate on the same narrow-bandgap semiconductor owing to the straddling band offset, thereby confirming that Type-I systems fail to achieve spatial charge separation regardless of interfacial contact equilibration [37]. Consequently, both electrons and holes accumulate on SC-II (electrons gather in the CB of SC-II and holes in the VB of SC-II). Because both carrier types concentrate within the same semiconductor component, charge recombination readily occurs in the bulk or at the interface, failing to achieve effective spatial separation [38].

### 3.2. Type-II Heterojunction

In a Type-II heterojunction (Figure 3b), the band alignments of SC-I and SC-II are staggered. Upon photoexcitation, electron–hole pairs are generated in both components [39]. Since the CB potential of SC-II is more negative (higher electron energy), photogenerated electrons in SC-II spontaneously migrate to the more positive (lower energy) CB of SC-I; simultaneously, photogenerated holes on the more positive VB of SC-I transfer to the more negative (higher energy) VB of SC-II [40]. In conventional discussions of Type-II heterojunctions, energy levels are frequently depicted using simplified flat-band alignments. Before contact, SC-I and SC-II feature staggered band edges with different Fermi levels. Upon physical contact, free electrons migrate from the component with the higher Fermi level (lower work function) to that with the lower Fermi level, creating an interfacial internal electric field (IEF) and inducing upward/downward band bending at the junction boundary. Steady-state photogenerated electrons in the higher CB transfer to the lower CB, while holes migrate in the opposite direction. Explicitly acknowledging this contact-induced band bending clarifies that the bidirectional migration in Type-II junctions operates in competition with the interfacial barrier, which partially accounts for the thermodynamic driving force loss commonly observed in real systems [41].

Consequently, photogenerated electrons collect on the CB of SC-I, whereas holes accumulate on the VB of SC-II. Therefore, Type-II heterojunctions retain electrons with a weaker reduction potential on SC-I and holes with a weaker oxidation potential on SC-II, representing an inherent thermodynamic limitation [42]. Before contact, isolated SC-I and SC-II exhibit independent band edges and work functions. Upon intimate interfacial contact, spontaneous electron transfer occurs driven by the Fermi-level difference until equilibrium is established at the interface, generating band bending and an interfacial contact barrier. Under subsequent illumination, photogenerated electrons slide down the staggered conduction band offset, while holes transfer along the valence band gradient.

### 3.3. Z-Scheme Heterojunction

In an all-solid-state direct Z-scheme heterojunction (Figure 3c), the semiconductor with deeper band positions (e.g., BiOBr, Fe_2_O_3_) is typically denoted as SC-I, while the more negative component (e.g., g-C_3_N_4_) is designated as SC-II [43]. As systematically formulated by Low et al. [37] in an all-solid-state direct Z-scheme heterojunction, staggered band alignment is retained while inactive carriers undergo interfacial recombination, thereby preserving strong redox overpotentials on the outer band edges. Under visible illumination, both components are excited to generate electron–hole pairs. Subsequently, photogenerated electrons with weaker reduction ability in the CB of SC-I migrate toward the interface and recombine rapidly with photogenerated holes with weaker oxidation ability in the VB of SC-II [44]. Through this directional recombination process, high-energy electrons (e^−^) with strong reduction potential are preserved in the CB of SC-II, migrating to the surface to drive O_2_^•−^ formation or participate directly in reductive dehalogenation/defluorination [45]; simultaneously, deep-energy holes (h^+^) with strong oxidation capacity remain in the VB of SC-I to directly oxidize organics or generate ·OH for deep ring-cleavage reactions [46,47,48]. Before contact, the two semiconductors exhibit staggered band structures. Following interfacial contact, charge redistribution forces their respective Fermi levels into alignment, generating localized band bending and an interfacial contact barrier. Concurrently, the accumulated charge density and localized interface states at the junction facilitate the quasi-ohmic recombination of relatively low-potential electrons from the CB of SC-I with low-potential holes from the VB of SC-II. Consequently, the highly reductive electrons in SC-II and strongly oxidative holes in SC-I are preserved at the outer spatial regions, resolving the apparent thermodynamic paradox [49]. This pathway integrates efficient spatial carrier separation with the maximum preservation of high redox potentials, cleverly overcoming the intrinsic thermodynamic sacrifice of traditional Type-II heterojunctions [50,51,52].

### 3.4. S-Scheme (Step-Scheme) Heterojunction

An S-scheme heterojunction (Figure 3d), first introduced by Fu et al. [53] to describe directional charge transfer between an oxidation photocatalyst (OP) and a reduction photocatalyst (RP) with staggered band alignment, represents a conceptual evolution beyond the traditional Z-scheme model [54]. In the dark, electrons spontaneously diffuse from SC-II to SC-I until Fermi levels equilibrate [55], causing SC-II to become positively charged and SC-I negatively charged [56]. This forms an interfacial built-in electric field (IEF) directed from SC-II toward SC-I and drives downward band bending in SC-I alongside upward band bending in SC-II [57,58]. Under photoexcitation, the synergy of the IEF, band bending potential barriers, and Coulombic attraction drives electrons with lower reduction potential in the CB of SC-I to recombine preferentially with lower-oxidation-potential holes in the VB of SC-II at the interface [59,60]; meanwhile, highly reductive high-energy electrons and highly oxidative high-energy holes are preserved in the CB of SC-II (RP) and the VB of SC-I (OP), respectively [61]. This mechanism accomplishes efficient spatial carrier separation while maximally maintaining the robust redox overpotentials of the system.

### 3.5. Experimental Criteria for Verifying Heterojunction Charge-Transfer Pathways

Mechanistic assignments of type-II, Z-scheme, and S-scheme pathways should not rest on thermodynamic band-alignment estimates alone, as staggered band structures are compatible with multiple charge-transfer routes. A robust assignment generally combines several complementary experiments: (i) band-edge and Fermi-level determination, using valence-band XPS or ultraviolet photoelectron spectroscopy together with Mott–Schottky analysis and UV–vis diffuse reflectance, to establish band positions and work functions of both components before and after contact [62]; (ii) Kelvin probe force microscopy or surface photovoltage measurements, which map interfacial potential differences and the direction of the built-in electric field [63]; (iii) in situ irradiated XPS, in which light-induced binding-energy shifts monitor interfacial electron redistribution; however, such shifts should not be treated as a stand-alone direct proof of an S-scheme mechanism due to potential surface photovoltage artifacts or differential charging. A definitive assignment requires cross-validation with work-function/Fermi-level alignments (UPS/Kelvin probe), built-in electric field direction (SPV/KPFM), and time-resolved carrier-lifetime dynamics [64]; (iv) time-resolved spectroscopy, such as femtosecond transient absorption and time-resolved photoluminescence, which resolves carrier lifetimes and interfacial transfer kinetics [65]; and (v) reactive-species profiling, combining electron paramagnetic resonance spin trapping, radical-quenching experiments, and probe-molecule assays, to verify that the redox products actually formed are thermodynamically consistent with the proposed pathway [66]. Cross-validation with at least two independent techniques is recommended before a charge-transfer mechanism is claimed.

## 4. Preparation Methods of g-C_3_N_4_-Based Heterojunctions

Two primary factors govern the catalytic efficiency of carbon nitride heterojunctions: selecting secondary semiconductors with suitable band matching to govern carrier migration and maintain redox capacity, and employing effective heterojunction construction methods. Protocols including chemical vapor deposition, hydrothermal/solvothermal methods, high-temperature calcination, sol–gel processing, in situ polymerization and deposition–precipitation and electrostatic/ultrasonication assembly and photodeposition have been successfully utilized [67], a comprehensive comparison of which is summarized in Table 1.

### 4.1. Chemical Vapor Deposition (CVD)

Chemical vapor deposition vaporizes solid or liquid precursors at elevated temperatures and transports the vapor phase via carrier gases into a high-temperature reaction zone, where decomposition, reaction, or polymerization occurs on substrate surfaces to yield thin-film materials. Under an inert atmosphere at elevated temperatures, vaporized organic precursors condense and polymerize in situ onto substrates or pre-synthesized g-C_3_N_4_ surfaces, facilitating the conformal growth of secondary semiconductors with atomic-scale interfacial bonding [68,69]. Chubenko et al. [70] reported g-C_3_N_4_ films grown by chemical vapor deposition on various substrates, providing a scalable route to uniform coatings. CVD represents a primary route for producing g-C_3_N_4_-based heterojunction films with high crystallinity, clean interfaces, and structural continuity [71].

### 4.2. Hydrothermal/Solvothermal Method

Hydrothermal and solvothermal methods are widely used to construct g-C_3_N_4_-based heterojunctions under relatively mild conditions—typically at operational temperatures of 120–200 °C and autogenous pressures below 2 MPa within sealed Teflon-lined autoclaves—compared to conventional high-temperature solid-state calcination (>500 °C). A representative case is the in situ hydrothermal growth of a g-C_3_N_4_–Bi_2_O_3_ heterojunction at 180 °C for 12 h in a Teflon-lined autoclave, in which the optimized composite degraded 93% of tetracycline hydrochloride within 90 min under UV-A irradiation [72]. These sealed, subcritical liquid environments enable precise control over crystallite nucleation, facet exposure, and intimate interfacial contact while preserving the thermal integrity of the polymeric melon network [73,74]. Moreover, by optimizing reaction temperatures and solvent properties, hydrothermal/solvothermal routes promote highly dispersed, intimate anchoring of metal oxides and other semiconductors across g-C_3_N_4_, exposing additional active sites while enhancing interfacial charge transfer efficiency [75].

### 4.3. High-Temperature Calcination

High-temperature calcination, also termed thermal polycondensation, involves blending g-C_3_N_4_ molecular precursors with secondary semiconductor precursors, followed by high-temperature processing [76]. During heating, nitrogen-rich precursors undergo deamination and condensation into g-C_3_N_4_, while the secondary component undergoes decomposition, oxidation, sulfidation, or crystallization, yielding the heterojunction in a single step. Compared to multi-step post-assembly routes, this method establishes stronger interfacial chemical bonding [77]. Zhang et al. [78] prepared a TiO_2_/g-C_3_N_4_ heterojunction by in situ growth of TiO_2_ nanocrystals on g-C_3_N_4_ nanosheets through an impregnation–calcination route, which produced intimate interfacial contact and enhanced charge separation. Thermal condensation of pure g-C_3_N_4_ typically occurs between 500 and 600 °C in covered alumina crucibles under air or inert gas at heating rates of 2–10 °C·min^−1^ for 2–4 h [79]. Excess secondary phases can lead to particle agglomeration or bury active sites, requiring careful optimization of precursor ratios, temperatures, and heating rates [80].

### 4.4. Sol–Gel Method

The sol–gel approach uses metal alkoxides or inorganic salts as precursors to form a uniform colloidal sol via hydrolysis and condensation, which transitions into a gel and converts to composite semiconductors after drying and heat treatment [81]. Its core advantage lies in achieving uniform mixing at molecular or nanoscale levels. Exfoliated g-C_3_N_4_ nanosheets are dispersed in an alcohol–water matrix to guide controlled hydrolysis and condensation of metal precursors across the sheets, forming core–shell or anchored heterostructures [82,83,84]. Zhang et al. [85] prepared g-C_3_N_4_/TiO_2_ heterostructured nanocomposites via a modified sol–gel route coupled with calcination. By slowly hydrolyzing tetrabutyl titanate in an ethanolic suspension containing exfoliated g-C_3_N_4_, ultrafine TiO_2_ nanoparticles were uniformly anchored onto g-C_3_N_4_ surfaces, markedly boosting specific surface area and interfacial charge separation while conferring superior activity and stability.

### 4.5. In Situ Polymerization and Deposition–Precipitation

In situ polymerization involves introducing pre-synthesized secondary semiconductors, coordination complexes, or monomer precursors directly into the polycondensation stage of carbon nitride precursors (e.g., urea, thiourea, melamine, or dicyandiamide) [86,87]. Under thermal treatment, the volatilized or condensing molecular precursors polymerize directly across the surface of the embedded guest components, forming an interpenetrated, tightly wrapped hybrid network [88,89]. This strategy avoids the macroscopic phase segregation and interfacial cracking often encountered during post-synthesis mechanical mixing, effectively establishing atomically intimate contact and extended pi-conjugated interfacial pathways that facilitate rapid charge transfer [90,91]. Deposition–precipitation utilizes wet-chemical precipitation to anchor secondary nanoparticles onto dispersed g-C_3_N_4_ nanosheets [92]. A precipitating agent (e.g., NaOH, NH_4_OH, or in situ urea hydrolysis) is slowly introduced into an aqueous suspension containing exfoliated g-C_3_N_4_ and metal cations under continuous stirring, driving the controlled, homogeneous nucleation and deposition of insoluble metal hydroxides or basic carbonates onto g-C_3_N_4_ active sites [93,94]. Subsequent low-temperature washing and mild calcination convert the precipitates into well-crystallized metal oxide or sulfide nanoparticles with high surface dispersion, minimizing severe nanoparticle self-aggregation [95,96].

### 4.6. Electrostatic/Ultrasonication Assembly and Photodeposition

Electrostatic and ultrasonication-assisted assembly is a facile, non-destructive physical assembly method governed by surface charge matching [97,98]. Because exfoliated g-C_3_N_4_ nanosheets typically possess negative surface zeta potentials across a wide pH range, they can spontaneously electrostatically assemble with positively charged secondary nanomaterials (e.g., layered double hydroxides, Bi-based oxyhalides, or modified metal oxides) in aqueous media under ultrasonic dispersion [99,100]. High-intensity ultrasound generates localized acoustic cavitation, promoting high-speed microjets that facilitate intimate 2D/2D facial contact without destroying the intrinsic crystal lattices or exposed reactive facets of the parent materials [101,102]. Photodeposition is an in situ, light-driven chemical assembly route conducted at ambient temperature [103]. Under visible or UV light irradiation, photogenerated electrons or holes that migrate to the g-C_3_N_4_ surface selectively reduce metal ions (e.g., Ag^+^, Pt^4+^, Au^3+^, Pd^2+^) into metallic nanoparticles or oxidize metal ions (e.g., Mn^2+^, Co^2+^) into metal oxides [104,105]. Because the growth of the secondary phase occurs strictly at the photogenerated charge accumulation sites, photodeposition yields precise, facet-selective nanoscale heterojunctions with minimal interfacial defects and low energy consumption [106,107].

**Table 1:** Comprehensive comparison of mainstream and emerging fabrication strategies for g-C_3_N_4_-based heterojunctions.

Fabrication Route	Typical Conditions (Temp./Pressure)	Interfacial Bonding Nature	Morphology Control	Energy Demand	Scalability	Key Advantages	Major Limitations	Ref.
Chemical Vapor Deposition (CVD)	500–650 °C; inert atmosphere or vacuum	Atomic-scale, continuous, pinhole-free contact	High (uniform conformal thin films)	High	Moderate	Clean interface, minimal defects, superior structural stability	High equipment cost; low yield for bulk powdered photocatalysts	[70]
Hydrothermal/Solvothermal	120–200 °C; 0.2–2 MPa (autogenous)	Strong chemical coordination/covalent bonds	High (in situ vertical nucleation and growth)	Moderate	High	Intimate interfacial contact, tailored crystal facets, high dispersion	Uses organic solvents; autoclave volume and safety limits	[72]
High-Temperature Calcination	500–550 °C; air or inert gas	Robust covalent/metalloid interfacial bonds	Moderate (risk of thermal sintering)	High	Very high	Simple one-step route, solvent-free, highly scalable	Thermal agglomeration; potential burial of surface active sites	[78,79]
Sol–Gel Method	25–100 °C (followed by mild annealing)	Molecular-level anchorages and coordination	High (core–shell or nano-anchored structures)	Moderate	High	Uniform precursor dispersion, high specific surface area	Susceptible to cracking during drying; requires multi-step calcination	[82,83,84,85]
In Situ Polymerization	80–180 °C; wet/hydrothermal conditions	Interpenetrated π-conjugated network	Good (intercalated or wrapped architectures)	Low to moderate	High	Prevents phase segregation; preserves exposed active facets	Unreacted precursor residues require rigorous extraction	[88,91]
Electrostatic/Ultrasonication Assembly	Room temperature (20–50 °C); ambient pressure	Interfacial Coulombic forces and van der Waals	Moderate (governed by precursor morphology)	Very low	High	Facile, green, room-temperature operation, non-destructive	Weaker interfacial binding; risk of particle detachment in continuous flow	[101,102]
Photodeposition	20–40 °C; ambient pressure under light	Selective facet-dependent deposition	High (targeted localized nanoparticle loading)	Low	Moderate	Room-temperature, site-selective co-catalyst anchoring	Restricted to light-reducible or light-oxidizable metal ions	[104,106]

## 5. Applications of g-C_3_N_4_ Heterojunctions in Water Purification

Through band-alignment engineering and accelerated charge-carrier separation, g-C_3_N_4_-based heterojunctions resolve key limitations of pristine g-C_3_N_4_, including rapid carrier recombination, inadequate redox potentials, and restricted visible-light absorption. Recent progress in photocatalytic water purification is reviewed below across emerging contaminant degradation, pathogenic microbial inactivation, and high-valence heavy metal reduction [108]. To offer a comprehensive perspective, representative g-C3N4-based heterojunction photocatalytic systems across these three water purification domains—including their heterojunction types, primary reactive species, operational parameters, and target pollutant removal efficiencies—are systematically summarized in Table 2.

### 5.1. Degradation of Emerging Contaminants

**Figure 4:**
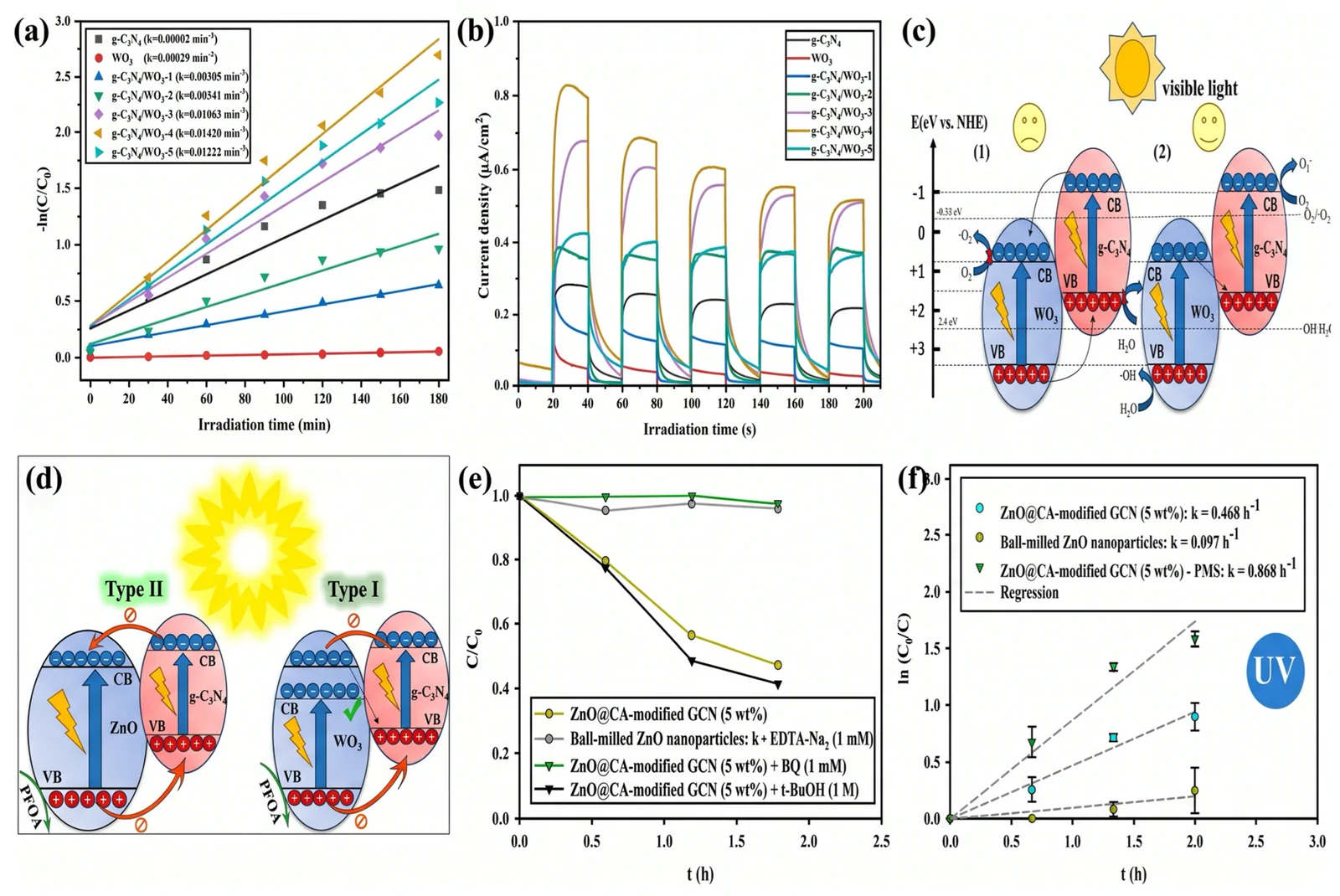
Photocatalytic degradation performance, interfacial charge transfer and mechanism validation of g-C_3_N_4_-based heterojunctions. (**a**) Pseudo-first-order kinetic plots of pollutant degradation over g-C_3_N_4_/WO_3_ composites with different mass ratios; (**b**) transient photocurrent responses of pristine g-C_3_N_4_, pure WO_3_ and g-C_3_N_4_/WO_3_ heterojunctions; and (**c**) schematic illustration of charge migration pathways and reactive oxygen species generation over the g-C_3_N_4_/WO_3_ heterojunction under visible light. Reproduced from Ref. [116] under CC BY 4.0 license; (**d**) Schematic illustration comparing the charge transfer mechanisms of Type-II and Type-I heterojunctions construct-ed from ZnO and N-vacancy-rich CA-modified GCN under light irradiation. Green arrows with checkmarks (✔) denote the thermodynamically favored charge transfer pathways and the PFOA degradation routes driven by pho-togenerated holes. Orange curved arrows accompanied by red prohibition signs (⊘) indicate the thermodynamically unfavorable or obstructed pathways. The labels e^−^ and h^+^ represent photogenerated electrons and holes in the re-spective conduction and valence bands; (**e**) effects of different radical scavengers on the photocatalytic activity of ZnO@CA-modified g-C_3_N_4;_ and (**f**) kinetic comparison of pollutant degradation over different catalytic systems under UV irradiation. Reproduced from Ref. [120] under CC BY 4.0 license.

Emerging aquatic contaminants, such as microplastics (MPs), antibiotics, persistent organic pollutants (POPs), and endocrine-disrupting chemicals (EDCs), exhibit persistent environmental resistance and high ecotoxicity [109,110,111]. Constructing g-C_3_N_4_-based heterojunctions synergistically enhances adsorption capacity and carrier dynamics, supplying conduction-band electrons and reactive oxygen species (ROS) to drive dehalogenation, aromatic ring opening, and thorough mineralization. Pristine g-C_3_N_4_ displays limited activity toward recalcitrant microplastics, typically yielding mass loss below 15% without measurable mineralization. In evaluating microplastic removal, it is imperative to clearly delineate physical adsorption, mechanical fragmentation, surface oxidation, macromolecular chain scission, mass loss, and authentic TOC mineralization to avoid overestimating degradation efficacy. For instance, physical adsorption concentrates microplastics onto the catalyst surface, whereas true photocatalytic destruction is initiated by reactive radical attack on polymer backbones, inducing oxygen-containing functional groups (C–O, C=O), successive chain scission into short-chain oligomers, and ultimate mineralization into CO_2_ and H_2_O [112,113]. Nguyen et al. [114] synthesized a TiO_2_-modified UiO-66/g-C_3_N_4_ heterojunction from terephthalic acid recovered from waste PET, which achieved up to 90% visible-light degradation of polystyrene microplastics within 30 h; electrochemical impedance spectroscopy confirmed efficient interfacial charge transfer. Jia et al. [115] fabricated oxygen-vacancy-rich MoO_3-x_/g-C_3_N_4_ Z-scheme heterojunctions, achieving 97% degradation under continuous visible illumination for 7 days, whereas pristine g-C_3_N_4_ remained largely inactive. The heterojunction exhibited superior carrier separation and transport kinetics; its direct Z-scheme alignment was strictly substantiated by in situ irradiated XPS (displaying binding energy shifts of Mo 3d and C 1s toward opposite directions under light) and DMPO-trapping EPR, which confirmed the simultaneous generation of both O_2_^•−^ and ·OH without sacrificial hole consumption while preserving high redox overpotentials. Lu et al. [116] designed a direct Z-scheme g-C_3_N_4_/WO_3_ nanocomposite, achieving a 93.3% photodegradation of tetracycline hydrochloride (TC-HCl) under visible light with an apparent reaction rate constant (*k* = 0.0142 min^−1^) substantially surpassing pristine counterparts (Figure 4a). The transient photocurrent responses (Figure 4b) revealed a substantially stronger photocurrent for the g-C_3_N_4_/WO_3_ heterojunction than for pristine g-C_3_N_4_ and pure WO_3_, indicating more efficient separation and interfacial transfer of photogenerated electron-hole pairs. Crucially, spin-trapping ESR spectra explicitly resolved the concurrent evolution of robust DMPO-O_2_^•−^ and DMPO-·OH signals under irradiation. Because the single-phase band potentials of WO_3_ conduction band and g-C_3_N_4_ valence band are thermodynamically incapable of directly yielding O_2_^•−^ and ·OH, respectively, this dual-ROS generation provided unequivocal spectroscopic verification for the direct Z-scheme charge-transfer pathway (Figure 4c), which spatially segregates reactive carriers while preserving high redox overpotentials. Electron paramagnetic resonance (EPR) spin trapping and radical scavenging assays confirmed that high concentrations of photogenerated O_2_^•−^ and ·OH drove the oxidative cleavage, while liquid chromatography–mass spectrometry (LC–MS) tracked the transformation intermediates, confirming the sequential breakdown of macromolecular chains and final detoxification.

Heterojunction architectures demonstrate outstanding efficiency in antibiotic mineralization and antibiotic resistance gene (ARG) disruption [117,118]. Zheng et al. [119] prepared a g-C_3_N_4_/Cr_2_O_3_ composite governed by an S-scheme charge transfer mechanism, retaining high-reduction electrons in the g-C_3_N_4_ CB and high-oxidation holes in the Cr_2_O_3_ VB. Under this synergy, 63.8% of tetracycline TOC mineralization was achieved within 50 min, with a rate constant 2.6 times that of pristine g-C_3_N_4_. For POPs with high bond energies (e.g., C–F and C–Cl bonds), heterojunctions continuously supply energetic electrons and ROS to drive deep dehalogenation and mineralization. Parallel efforts tackling POPs have focused on per- and polyfluoroalkyl substances (PFAS), where the exceptional bond dissociation energy of C–F linkages (~485 kJ/mol) resists conventional degradation pathways. Navidpour et al. [120] constructed a ZnO@citric acid-modified g-C_3_N_4_ hybrid catalyst capable of accelerated perfluorooctanoic acid (PFOA) elimination (Figure 4d). The optimized 5 wt% hybrid exhibited an apparent rate constant of 0.468 h^−1^ under UV light (a 4.8-fold improvement over ball-milled ZnO), which was further elevated to 0.868 h^−1^ upon peroxymonosulfate (PMS) activation (Figure 4e); Scavenger quenching assays and charge-transfer modeling confirmed that photogenerated holes (h^+^) alongside derived O_2_^•−^ and SO_4_^•−^ radicals trigger primary Kolbe decarboxylation (C_7_F_15_COO^−^→ C_7_F^•^_15_ + CO_2_), subsequently initiating sequential chain-shortening cycles that drive comprehensive defluorination into fluoride ions and CO_2_ (Figure 4f). Kang et al. [121] synthesized an oxygen-vacancy-rich direct Z-scheme g-C_3_N_4_/BiOBr heterojunction via a PVP-assisted solvothermal approach. The optimal composite (CN/VoBOB-15, 15 wt% g-C_3_N_4_) achieved 93% RhB and 91% Congo red degradation within 30 min under LED irradiation, yielding an apparent rate constant (*k* = 0.07667 min^−1^ for RhB) that was 57.6 and 6.7 times higher than those of pristine g-C_3_N_4_ and vacancy-rich BiOBr, respectively. Active-species trapping and DFT work-function evaluations confirmed a direct Z-scheme charge-transfer mechanism, driven by synergistic carrier separation and retained strong redox overpotentials (O_2_^•−^ and h^+^ dominant). When treating EDCs containing stable aromatic structures and phenolic groups [122,123], complex water matrices demand elevated catalytic durability. Duan et al. [124] fabricated an intimate 2D/2D direct Z-scheme g-C_3_N_4_/Bi_2_O_2_CO_3_/porous g-C_3_N_4_ heterojunction via a two-stage template-free route. The optimized composite with 2 wt% Bi_2_O_2_CO_3_ exhibited a pseudo-first-order rate constant of *k* = 0.0608 min^−1^ toward tetracycline hydrochloride under simulated sunlight, which was 5.1 times higher than that of porous g-C_3_N_4_ nanosheets, achieving 97.4% degradation within 60 min with robust stability over five consecutive cycles. Masekela et al. [125] reviewed MXene-based nanocomposites for photocatalytic wastewater treatment, CO_2_ reduction, and H_2_ production, highlighting g-C_3_N_4_/MXene heterojunctions as a promising family for enhanced charge separation. Zhao et al. [126] developed a Bi-based ternary heterojunction that maintained over 78% degradation efficiency in complex real water matrices, demonstrating practical application potential. In addition to refractory antibiotics and emerging POPs, classic organic dye degradation remains an essential benchmark for evaluating carrier transfer efficiency. Dai et al. [127] constructed a 5 wt% Co_3_O_4_/g-C_3_N_4_ Type-II heterojunction via coupled hydrothermal and calcination methods. Driven by the staggered band alignment, photogenerated electrons accumulated on the g-C_3_N_4_ conduction band while holes were steered to the Co_3_O_4_ valence band, delivering a Rhodamine B (RhB) degradation rate of 97.6% within 40 min under simulated solar light (*k* = 0.0703 min^−1^, 2.93- and 5.58-fold higher than pristine g-C_3_N_4_ and pure Co_3_O_4_, respectively). Active species trapping and ESR spin-trapping confirmed that O_2_^•−^ and h^+^ served as the dominant reactive entities governing the photooxidation process. A comparative appraisal across the heterojunction categories reveals that while Type-II systems provide directional electron/hole separation, their photocatalytic redox driving forces are inherently compromised because electrons and holes migrate to thermodynamically weaker levels. In contrast, all-solid-state direct Z-scheme and S-scheme configurations preserve uncompromised redox potentials (e.g., highly negative CB electrons for O_2_^•−^ generation and deep VB holes for ·OH evolution), yielding noticeably superior mineralization rates (*k* values several-fold higher—typically 2–5-fold among the studies summarized here) and deeper dehalogenation capability toward refractory contaminants such as microplastics. From a practical water-treatment perspective, the presence of natural organic matter (NOM) in authentic surface waters inevitably induces active-site competition and light attenuation, while coexisting anions (HCO_3_^−^, Cl^−^, SO_4_^2−^) act as scavenger sinks for ·OH and h^+^, frequently resulting in lower reaction rates than in deionized water (commonly by ~15–30% in the cited studies).

### 5.2. Inactivation of Pathogenic Microorganisms

Pathogenic microorganisms in aquatic environments, including Gram-positive/negative bacteria, viruses, fungi, and algae, induce infectious diseases and threaten public health. Chlorination can form hazardous halogenated disinfection byproducts, while ultraviolet (UV) treatment is subject to high energy consumption, light attenuation from turbidity, and bacterial photoreactivation, offering limited efficacy toward resistant species [128]. Forming intimate heterojunction interfaces redirects carrier transport and curbs electron–hole recombination, generating higher yields of reactive species including h^+^, O_2_^•−^, ·OH and ^1^O_2_. These active species attack bacterial cell walls, membranes, peripheral proteins, intracellular enzymes, and nucleic acids, causing membrane permeabilization, cytoplasmic leakage, metabolic cessation, and irreversible DNA/RNA oxidation, thereby realizing multi-target disinfection. For instance, Zhan et al. [129] engineered an AgBr/g-C_3_N_4_ direct Z-scheme heterojunction that achieved complete visible-light inactivation of 10^7^ CFU/mL *E. coli* within 90 min. The Z-scheme preserves the strongly reducing electrons of g-C_3_N_4_ and the strongly oxidizing holes of AgBr, sustaining the concurrent generation of O_2_^•−^ and ·OH, although natural organic matter (>0.5 mg/L) attenuates the disinfection efficiency. Beyond chemical radical oxidation, Zhang et al. [130] developed a metal-free UCN/RGO/SCN homojunction demonstrating broad-spectrum bactericidal efficiency (>99.8% against both *E. coli* and *S. aureus*) under visible-light irradiation (λ > 400 nm). The sharp edges of the embedded RGO physically pierced and disrupted bacterial cell envelopes, while the dual carbon nitride homojunction suppressed charge-carrier recombination to sustain elevated yields of reactive O_2_^•−^ and ·OH species.

**Figure 5:**
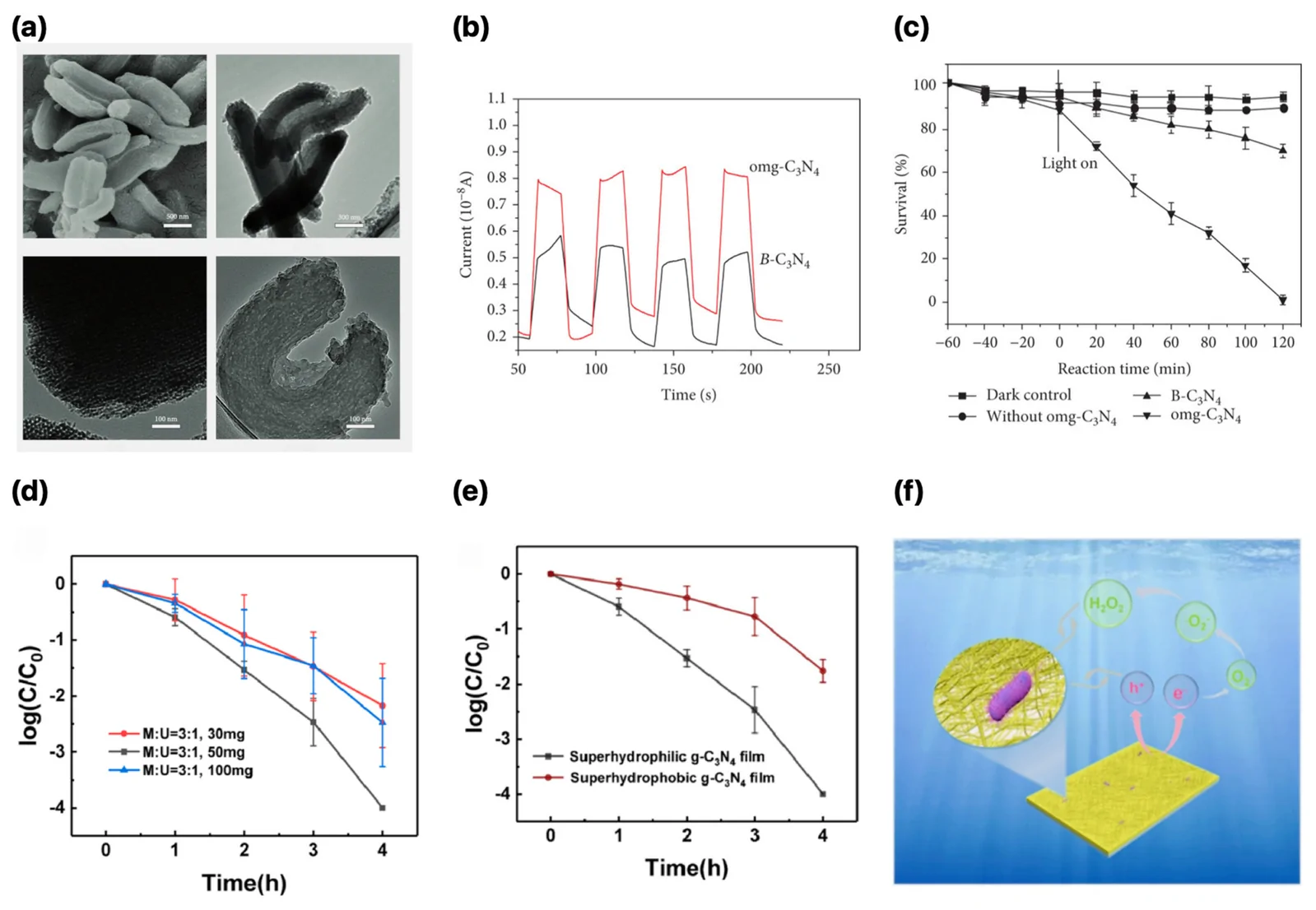
Visible-light-driven photocatalytic bacterial disinfection performance and mechanistic validation of g-C_3_N_4_-based photocatalysts: (**a**) SEM and TEM images of ordered mesoporous g-C_3_N_4_ (omg-C_3_N_4_); (**b**) transient photocurrent responses of omg-C_3_N_4_ and bulk g-C_3_N_4_ (B-C_3_N_4_) under repeated on–off visible-light illumination; and (**c**) bacterial survival curves obtained with omg-C_3_N_4_, B-C_3_N_4_, under dark conditions, and without catalyst. Reproduced from Ref. [131] under CC BY 4.0 license; (**d**) inactivation kinetics over g-C_3_N_4_ films prepared with different precursor masses (melamine:urea = 3:1; 30, 50 and 100 mg); (**e**) effect of surface wettability (superhydrophilic versus superhydrophobic film) on the disinfection performance; and (**f**) Schematic illustration of the proposed photocatalytic bacterial inactivation and reactive oxygen species (ROS) gen-eration mechanism over the catalytic film under light irradiation. Reproduced from Ref. [132] under CC BY 4.0 license.

In contrast to systems driven by diffused radicals, Chen et al. [131] synthesized ordered mesoporous g-C_3_N_4_ (omg-C_3_N_4_) using SBA-15 as a hard template, and SEM/TEM images show a worm-like morphology with hexagonally arranged mesopores (Figure 5a). The ordered mesoporous framework improves light harvesting and charge separation, as reflected in the substantially stronger transient photocurrent of omg-C_3_N_4_ than that of bulk g-C_3_N_4_ (B-C_3_N_4_) under repeated on–off illumination (Figure 5b). Accordingly, omg-C_3_N_4_ produced a much faster decline in bacterial survival than B-C_3_N_4_, the dark control, and the catalyst-free control (Figure 5c). Zhang et al. [132] further showed that the disinfection performance of a nanowired g-C_3_N_4_ film depends on the precursor mass used in the vapor-assisted confined deposition: at a fixed melamine:urea ratio of 3:1, the 50 mg sample outperformed the 30 and 100 mg samples (Figure 5d), while hydrophobic modification strongly suppressed inactivation relative to the superhydrophilic film (Figure 5e). Scavenger-quenching experiments identified H_2_O_2_ as the dominant reactive species (Figure 5f), indicating that H_2_O_2_, rather than ·OH, O_2_^•−^ or h^+^, drives the antibacterial action. In real disinfection systems, elevated water turbidity and bacterial aggregation impede photon penetration, while residual cellular fragments can foul catalyst interfaces. Establishing long-term bactericidal durability and confirming the absence of bacterial photoreactivation under ambient light post-treatment are vital for engineering applications.

### 5.3. Reduction of High-Valence Heavy Metal Ions

**Figure 6:**
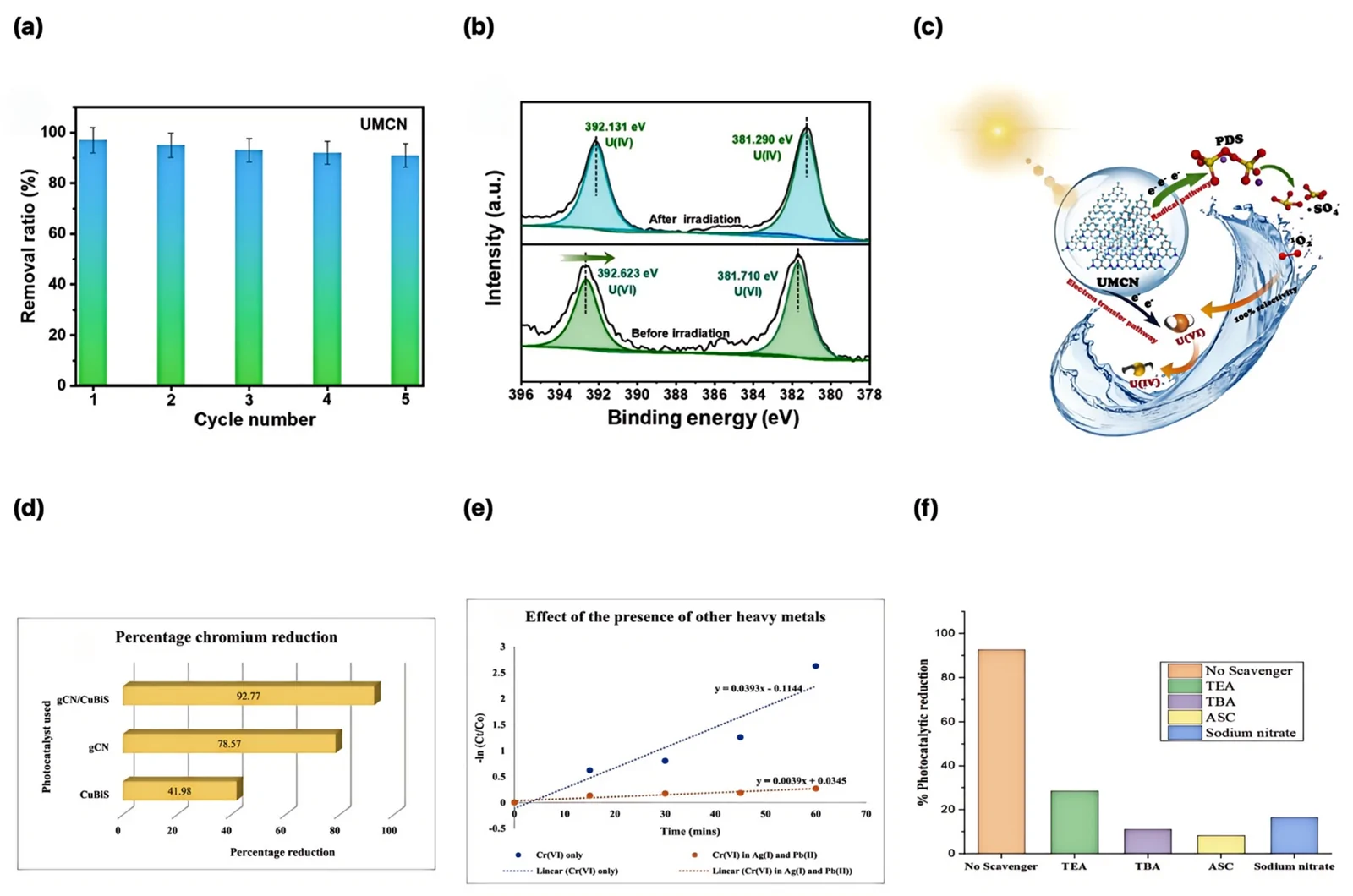
Photocatalytic reduction performance and mechanistic analysis of high-valence heavy metal ions over g-C_3_N_4_-based heterojunctions. (**a**) Reusability of UMCN, showing the U(VI) removal ratio over five successive cycling runs; (**b**) high-resolution U 4f XPS spectra recorded before and after irradiation, in which the U(VI) peaks at 381.710 and 392.623 eV shift to 381.290 and 392.131 eV, characteristic of U(IV); and (**c**) Schematic illustration of the proposed photocatalytic mechanism for selective U(VI) reduction over UMCN in the presence of PDS under light irradiation. The green arrow labeled "Radical pathway" indicates the transfer of photogenerated electrons to PDS to generate active radical species The black curved arrow labeled "Electron transfer pathway" represents the direct transfer of photogenerated electrons to the adsorbed U(VI) species. The yellow curved arrow with "100% selectivity" denotes the highly selective target attack, and the orange curved arrow signifies the reduction of high-valence soluble U(VI) to low-valence U(IV) precipitate. The blue sphere (left) represents UMCN with its embedded photoexcited electrons, while the top molecular models represent PDS activation products, and the bottom models indicate the U(VI) to U(IV) transformation. Reproduced from Ref. [142] under CC BY 4.0 license; (**d**) photocatalytic Cr(VI) reduction efficiency of pristine Cu3.21Bi4.79S9, pure g-C3N4 and Cu3.21Bi4.79S9/g-C3N4 nanocomposite and (**e**) effect of co-existing Ag(I) and Pb(II) ions on the photoreduction kinetics of Cr(VI); (**f**) radical scavenging results for determining the primary reactive species involved in Cr(VI) reduction. Reproduced from Ref. [143] under CC BY 4.0 license.

Industrial effluents often contain toxic and persistent heavy metals and radionuclides, including chromium Cr(VI), mercury Hg(II), and uranium U(VI) [133]. Conventional precipitation and adsorption approaches frequently suffer from excessive sludge generation and poor trace-level sequestration [134]. Heterojunction systems based on g-C_3_N_4_ exploit energetic conduction-band electrons to reduce mobile, high-valence species into low-toxicity, low-solubility precipitates, thereby facilitating wastewater purification and resource recovery [135,136,137]. Dai et al. [138] demonstrated that g-C_3_N_4_-based S-scheme heterojunctions enhance interfacial carrier separation and increase the reduction potential of CB electrons toward radionuclides. Mechanistically, a rigorous chemical distinction must be established between true photocatalytic reduction and photo-assisted precipitation during uranium extraction. Under anaerobic conditions in the presence of organic hole scavengers, high-reduction CB electrons can directly reduce soluble hexavalent U(VI) (e.g., UO_2_^2+^) to insoluble tetravalent U(IV) precipitates (such as UO_2_ nanocrystals) [139]. Conversely, in aerated aqueous environments without sacrificial agents, extraction occurs via photo-assisted precipitation: high-energy conduction-band electrons reduce dissolved O_2_ into O_2_^•−^ and subsequent H_2_O_2_, which subsequently complexes with uranyl ions to precipitate solid studtite (UO_2_)O_2_·2H_2_O onto the catalyst surface. In this latter pathway, uranium remains strictly in the hexavalent state (+6), representing a reactive crystallization route rather than a valence reduction process. Dai et al. [140] developed an S-scheme ZnIn_2_S_4_/g-C_3_N_4_ heterojunction for U(VI) extraction under ambient air without sacrificial agents. High-reduction electrons in the g-C_3_N_4_ CB activated dissolved O_2_ to generate O_2_^•−^ and H_2_O_2_, while the interfacial electric field maintained efficient charge separation. Surface analyses confirmed the precipitation of soluble U(VI) into insoluble (UO_2_)O_2_·2H_2_O on the catalyst surface. The composite retained over 85% U(VI) removal after five consecutive cycles in the presence of competing co-ions. Mechanistically, the proposed S-scheme pathway was rigorously corroborated via contact potential difference measurements (KPFM), confirming a 0.42 V Fermi-level gradient and directional built-in electric field, while transient absorption spectroscopy validated the preferential recombination of non-effective carriers across the staggered interface. Constructing heterojunctions over g-C_3_N_4_ maximally retains high-reduction conduction band electrons (E_CB_ ≈ −1.1 to −1.3 V vs. NHE), supplying formidable thermodynamic overpotentials to convert soluble high-valence ions into insoluble low-valence precipitates. For radionuclide remediation, Liu et al. [141] fabricated a Z-scheme g-C_3_N_4_/TiO_2_ composite that removed >99% of aqueous U(VI) within 25 min under UV/solar light (*k* = 0.2222 min^−1^, 56.97 times that of pristine g-C_3_N_4_). U 4f XPS confirmed the formation of U(IV) (doublets at 379.6 and 390.4 eV), and the Z-scheme preserves the strongly reducing g-C_3_N_4_ conduction-band electrons that drive (U(VI) → U(V) → U(IV)). Liu et al. [142] tuned the N-doped state of polymeric carbon nitride (UMCN) to enrich delocalized electrons that activate peroxydisulfate (PDS); with PDS and H_2_O_2_ coexisting, 100% U(VI) photoreduction was achieved within 20 min. The U(VI) removal ratio was essentially retained over five successive cycling runs (Figure 6a), while U 4f XPS confirmed genuine valence reduction rather than mere adsorption: the U(VI) peaks at 381.710 and 392.623 eV were shifted to 381.290 and 392.131 eV after irradiation, characteristic of U(IV) (Figure 6b). Radical-trapping experiments and theoretical calculations further indicated that PDS-derived sulfate radicals cooperate with photogenerated electrons through coupled radical and electron-transfer pathways (Figure 6c). Ajiboye et al. [143] constructed a ternary Cu_3.21_Bi_4.79_S_9_/g-C_3_N_4_ heterojunction achieving 92.77% visible-light photoreduction of Cr(VI) (*k* = 0.0393 min^−1^, a 5.17-fold acceleration over the pristine chalcogenide) (Figure 6d). Crucially, surface zeta-potential determinations illuminated why acidic conditions (pH 2) are indispensable: the positively charged catalyst surface (+12.95 mV) electrostatically attracts negatively charged chromate species (Cr_2_O_7_^2−^/HCrO_4_^−^), dramatically mitigating interfacial mass-transfer resistance. Furthermore, evaluating real water-matrix perturbations revealed vital engineering boundaries: unlike simple sacrificial scavengers (e.g., methanol) that accelerate reduction, coexisting recalcitrant aromatic pollutants (e.g., bisphenol) and competing heavy-metal cations (e.g., Pb^2+^ and Ag^+^) induced profound antagonistic effects, prolonging the reduction half-life by over ten-fold due to competitive active-site occupancy and parasitic electron consumption (Figure 6e). Radical quenching assays confirmed that free photogenerated electrons (e^−^) were the primary reductants governing the detoxification process (Figure 6f).

Similarly, Yang et al. [144] synthesized flower-like Bi_2_S_3_/g-C_3_N_4_ composites via a polyethylene glycol (PEG)-assisted route, reducing soluble Cr(VI) to less toxic Cr(III) under visible light. Guided by a direct Z-scheme pathway, high-energy electrons in the g-C_3_N_4_ CB (E_CB_ ≈ −1.63 V vs. NHE, as reported under the measurement conditions of Ref. [144]) crossed the activation overpotential to rapidly reduce Cr(VI) to Cr(III), while photogenerated holes and derived O_2_^•−^ drove the concurrent degradation of coexisting organic dyes. In an S-scheme g-C_3_N_4_/Bi_2_WO_6_ system, coexisting organic pollutants acted as hole scavengers to suppress carrier recombination, accelerating Cr(VI) reduction kinetics [145]. Concurrently, high-reduction electrons in the g-C_3_N_4_ CB (E_CB_ ≈ −1.1 to −1.63 V vs. NHE) offer a strong thermodynamic driving force to drive the reduction of Cr(VI) to Cr(III), as the reduction potentials of Cr(VI) couples (such as HCrO_4_^−^/Cr^3+^ at +1.35 V vs. NHE, and Cr_2_O_7_^2−^/Cr^3+^ at +1.33 V vs. NHE at pH 0) are highly positive. Furthermore, the speciation and redox thermodynamics of Cr(VI) are strongly pH-dependent: acidic conditions dramatically favor the reduction kinetics because protons participate directly in the electron-transfer process, shifting the equilibrium toward rapid reduction of HCrO_4_^−^, whereas alkaline conditions favor the less reactive CrO_4_^2−^ species [146].

**Table 2:** Representative performance of g-C_3_N_4_-based heterojunctions in photocatalytic water purification (compiled from the studies reviewed in Section 5).

Heterojunction (Mechanism)	Target Contaminant	Operational Conditions (Dosage, Pollutant Conc., pH, Matrix)	Irradiation Source & Wavelength	Reaction Time (h/min)	Kinetic Rate Constant (*k*)	Mineralization /Removal Efficiency	Cyclic Reusability	Ref.
MoO_3−x_/g-C_3_N_4_ (Direct Z-scheme)	Polyaniline microplastics (PANI-MPs)	Cat.: 0.40 g/L; PANI: 10 mg/L; pH = 6.8; ultrapure water	500 W Xe lamp (λ ≥ 420 nm)	7 d (168 h)	*k* = 0.48 d^−1^	97% degradation; oligomer fragmentation confirmed by LC-MS	High photostability over prolonged run	[115]
g-C_3_N_4_/WO_3_ (Direct Z-scheme)	Tetracycline hydrochloride (TC-HCl)	Cat.: 1.0 g/L; TC-HCl: 30 mg/L; pH = 4.4; aqueous solution	300 W Xe lamp (λ > 400 nm)	180 min	*k* = 0.01420 min^−1^ (17.7× pristine g-C_3_N_4_)	93.3% degradation; dual ROS (O_2_^•−^ and ·OH) verified by ESR	4 cycles (97.8% retention)	[116]
g-C_3_N_4_/Cr_2_O_3_ (S-scheme)	Tetracycline (TC)	Cat.: 0.20 g/L; TC: 10 mg/L; natural surface water/river water	300 W Xe lamp (λ ≥ 420 nm)	50 min	*k* = 0.03121 min^−1^ (5.71× pristine g-C_3_N_4_)	83.4% TC degradation; 63.8% TOC mineralization; phytotoxicity eliminated	5 cycles (high stability)	[119]
ZnO@CA-modified g-C_3_N_4_ (S-scheme)	Perfluorooctanoic acid (PFOA)	Cat.: 0.53 g/L; PFOA: 53 mg/L; optional PMS (0.53 g/L)	300 W Xe lamp	120 min	*k* = 0.468 h^−1^ (0.868 h^−1^ with PMS)	~59% degradation (~79% with PMS); stepwise defluorination into F^−^ & CO_2_	4 cycles (robust durability)	[120]
g-C_3_N_4_/BiOBr (S-scheme)	Methylene blue; Congo red; tetracycline	Cat.: 0.50 g/L; MB: 20 mg/L; CR: 20 mg/L; TC: 50 mg/L river water (pH 4–10)	300 W Xe lamp (λ > 420 nm)	90 min	*k*(MB) = 0.02689 min^−1^	90.8% MB; 99.2% CR; 89% TC	5 cycles (>80.2% retention)	[122]
g-C_3_N_4_/Bi_2_O_2_CO_3_ (Direct Z-scheme)	Tetracycline hydrochloride(TC-HCl)	Cat.: 1.0 g/L; TC-HCl: 44.4 mg/L (1 × 10^−4^ M); neutral pH	Simulated solar light (Xe lamp)	60 min	*k* = 0.0608 min^−1^ (5.1× porous g-C_3_N_4_))	97.4% degradation within 60 min	5 cycles (no distinct loss)	[124]
Co_3_O_4_/g-C_3_N_4_ (p-n/Type-II)	Rhodamine B (RhB)	Cat.: 0.20 g/L; RhB: 10 mg/L; aqueous matrix	Simulated solar light (300 W Xe lamp)	40 min	*k* = 0.0703 min^−1^ (2.93× pristine g-C_3_N_4_)	97.6% degradation; O_2_^•−^ and h^+^ dominant	3 cycles (no significant loss)	[127]
AgBr/g-C_3_N_4_ (Direct Z-scheme)	*Escherichia coli* (*E. coli*)	Cat.: 0.10 g/L; 10^7^ CFU/mL; pH = 7.0; saline solution (0.85% NaCl)	300 W Xe lamp (λ > 420 nm)	90 min	*k* = 0.078 min^−1^	100% disinfection (complete cellenvelope destruction & cytoplasmic leakage)	4 cycles (>99.9% bactericidal rate)	[129]
UCN/RGO/SCN (Homojunction)	*E. coli* & *S. aureus*	Cat.: 1.0 g/L; 10^7^ CFU/mL; saline solution	300 W Xe lamp (λ > 400 nm)	180 min	*k* ≈ 0.0392 min^−1^	99.92% (*E. coli*); 99.85% (*S. aureus*)	5 cycles (sustained activity)	[130]
ZnIn_2_S_4_/g-C_3_N_4_ (S-scheme)	Uranium extraction (U(VI))	Cat.: 0.20 g/L; U(VI): 50 mg/L; pH = 5.0; air atmosphere; natural water	300 W Xe lamp (λ > 420 nm)	480 min	*k* = 0.015 min^−1^	>94% removal via reactive surface precipitation as (UO_2_)O_2_·2H_2_O	5 cycles (>85% extraction retained)	[140]
Bi_2_S_3_/g-C_3_N_4_ (Direct Z-scheme)	Cr(VI) & Rhodamine B (RhB)	Cat.: 0.50 g/L; Cr(VI): 20 mg/L, RhB: 10 mg/L; pH = 2.0; acidic wastewater	300 W Xe lamp (λ > 420 nm)	30 min	*k* = 0.228 min^−1^ (Cr(VI) reduction)	100% Cr(VI) reduction to Cr(III); simultaneous 96% RhB decolorization	4 cycles (>90% reduction efficiency retained)	[144]

**Figure 7:**
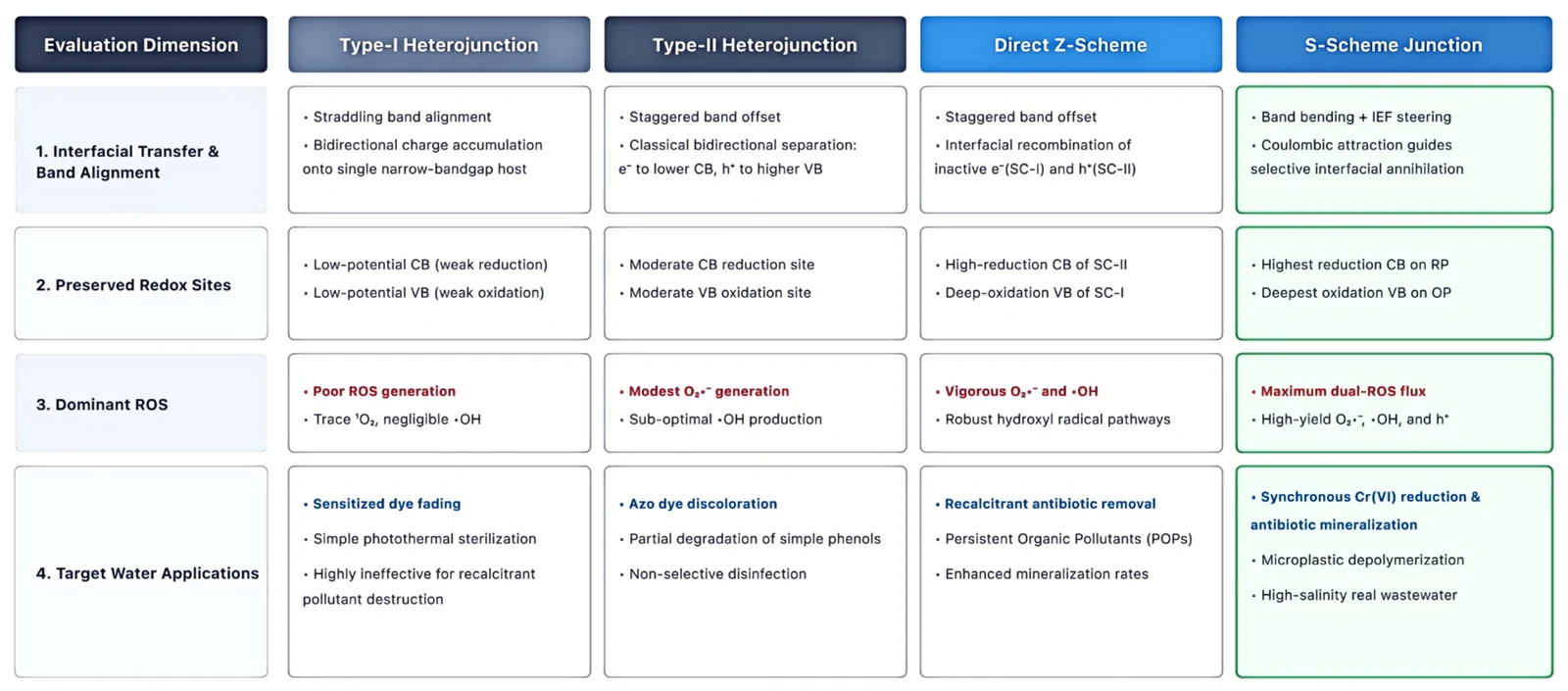
Schematic summary correlating heterojunction classifications (Type-I, Type-II, direct Z-scheme, and S-scheme) with their interfacial charge-transfer dynamics, preserved redox reaction centers, dominant reactive oxygen species (ROS), targeted aquatic decontamination domains, and respective limitations.

To synthesize the interfacial physics, reactive species generation, and technological trade-offs discussed across preceding sections, Figure 7 illustrates a holistic conceptual framework connecting heterojunction configurations (Type-I, Type-II, direct Z-scheme, and S-scheme) to their respective carrier transfer trajectories, preserved redox-active sites, dominant reactive oxygen species (ROS) and representative water remediation targets. This holistic author-generated synthesis provides an actionable decision-making matrix for tailoring g-C_3_N_4_-based architectures toward specific environmental decontamination challenges.

## 6. Conclusions and Perspectives

This review systematically summarized the structural features, synthetic methods, and environmental applications of g-C_3_N_4_-based heterojunctions in photocatalytic water purification. While g-C_3_N_4_ exhibits favorable band positions, non-toxicity, and stability, pristine formulations are hindered by rapid carrier recombination, narrow optical absorption, and low active-site exposure. Heterojunction construction, particularly Z-scheme and S-scheme configurations, spatially separates electron–hole pairs while preserving strong redox driving forces, establishing effective platforms for emerging pollutant degradation, microbial disinfection, and toxic metal reduction.

To translate laboratory-scale g-C_3_N_4_ heterojunction photocatalysis into engineering practice, future research must address five key dimensions:

(1) Real-World Water Matrix Resilience and Selective Interfacial Catalysis: The vast majority of reported studies evaluate degradation kinetics in deionized water, masking critical matrix effects encountered in municipal and industrial effluents. Future work must routinely benchmark photocatalysts against ubiquitous natural organic matter (NOM, e.g., humic and fulvic acids) that block active sites and light penetration, as well as background inorganic anions (HCO_3_ ^−^, Cl^−^, SO_4_^2−^ and NO_3_^−^) that act as radical scavengers. Engineering surface hydrophilicity/charge repulsion and creating molecularly imprinted or size-selective catalytic interfaces are vital strategies to resist fouling and preserve selective contaminant destruction.
(2) Macroscopic Shaping, Catalyst Immobilization, and Continuous Membrane Reactors: Post-treatment separation and recovery of sub-micron suspension powders impose prohibitive energy and capital costs. Research should shift aggressively from loose powder systems to macroscopically engineered architectures. Promising directions include: (i) 3D monolithic shaping via direct ink writing or additive manufacturing; (ii) durable immobilization onto flow-through porous substrates (e.g., polyurethane sponges, ceramic foams, stainless-steel meshes, and optical-fiber arrays); and (iii) integration into continuous photocatalytic membrane reactors (PMRs) with submerged or self-cleaning filtration modules to prevent catalyst loss.
(3) AI-assisted Rational Design: Empirical trial-and-error synthesis of heterojunction interfaces must be replaced by data-driven predictive paradigms. Future research should combine first-principles hybrid-functional DFT calculations (evaluating interfacial binding energy, planar work functions, and charge density redistribution) with machine learning (ML) algorithms. Constructing standardized, high-quality feature libraries of component band alignments, surface termination states, and interfacial charge injection kinetics will enable accelerated screening of stable, highly active heterojunction candidate pairs prior to laboratory synthesis.
(4) Multi-Physics CFD Modeling and Full-Scale Photoreactor Design: Laboratory assessments using small batch beakers under perpendicular top-irradiation fail to represent fluid-mechanical and optical realities. Future engineering efforts must utilize computational fluid dynamics (CFD) coupled with radiative transfer models (RTM) to simulate local volumetric light absorption, turbulent fluid mixing, and substrate mass-transfer boundary layers. Developing continuous-flow, scalable solar reactor topologies—such as compound parabolic concentrators (CPCs), spinning disc reactors, and tubular thin-film flow configurations—is critical to maximizing photonic efficiency per unit footprint.
(5) Comprehensive Toxicological Profiling and Life-Cycle Assessment (LCA): Complete pollutant disappearance (decolorization) frequently generates persistent or more toxic transformation intermediates (e.g., hydroxylated or chlorinated derivatives). Subsequent evaluations must rigorously quantify total organic carbon (TOC) mineralization and couple high-resolution mass spectrometry (LC-QTOF-MS/MS) with in vivo and in vitro ecotoxicity screenings (e.g., Vibrio fischeri microtoxicity and zebrafish embryo assays). Concurrently, cradle-to-grave life-cycle assessments (LCA) and techno-economic analyses (TEA) must be standardized, reporting electrical energy per order (E_EO_) and carbon emissions to prove tangible advantages over conventional advanced oxidation processes (AOPs).

## Data Availability

No new data were created or analyzed in this study. Data sharing is not applicable to this article.
